# Impact on patients with oral squamous cell carcinoma in different anatomical subsites: a single-center study in Taiwan

**DOI:** 10.1038/s41598-021-95007-5

**Published:** 2021-07-29

**Authors:** Nan-Chin Lin, Su-I Hsien, Jui-Ting Hsu, Michael Y. C. Chen

**Affiliations:** 1 School of Dentistry, China Medical University, Taichung, Taiwan; 2 Department of Oral and Maxillofacial Surgery, Changhua Christian Hospital, Changhua, Taiwan; 3 Department of Oral and Maxillofacial Surgery, Show Chwan Memorial Hospital, Changhua, Taiwan; 4 Department of Bioinformatics and Medical Engineering, Asia University, Taichung, Taiwan; 5 Dental Department and Division of Oral Maxillofacial Surgery, China Medical University Hospital, Taichung, Taiwan

**Keywords:** Cancer therapy, Head and neck cancer, Oral cancer

## Abstract

The incidence of oral cavity squamous cell carcinoma (OSCC) is particularly high in South Asia. According to the National Comprehensive Cancer Network, OSCC can arise in several subsites. We investigated survival rates and the clinical and pathological characteristics of OSCC in different anatomical subsites in the Taiwanese population. We retrospectively analyzed data for 3010 patients with OSCC treated at the Changhua Christian Hospital. Subsequently, we compared clinical and pathological features of OSCC in different subsites. Pathological T4 stage OSCCs occurred in the alveolar ridge and retromolar trigone in 56.4% and 43.7% of cases, respectively. More than 25% of patients with tongue OSCC and 23.4% of those with retromolar OSCC had lymph node metastasis. The prognosis was worst for hard palate OSCC (hazard ratio 1.848; *p* < 0.001) and alveolar ridge OSCC (hazard ratio 1.220; *p* = 0.017). Retromolar OSCC recurred most often and tongue OSCC second most often. The risk for cancer-related mortality was highest for hard palate OSCC, followed by alveolar ridge and retromolar OSCC. We found distinct differences in survival among the different subsites of OSCC. Our findings may also help prompt future investigations of OSCC in different subsites in Taiwanese patients.

## Introduction

Oral cavity cancer is the 11th most common malignancy in the world^1^ and causes many significant health problems^2^. The most common histological type of oral cavity cancer is squamous cell carcinoma, which accounts for more than 90% of cases^3^. Oral cavity squamous cell carcinoma (OSCC) is the sixth most common cancer in the world, and the incidence is particularly high in South Asia^4^. In Taiwan, it is currently the fourth and the seventh most commonly occurring malignant tumor in males and in both sexes, respectively^5^. In Taiwan, the buccal mucosa is the dominant subsite of OSCC; this fact could be ascribed to the common chewing of betel nuts^6–9^.

The National Comprehensive Cancer Network (NCCN) classifies the anatomic subsites of OSCC as the buccal mucosa, alveolar ridge, tongue, hard palate, retromolar trigone, floor of the mouth (FOM), and mucosa of the lips^10,11^. Lymphatic drainage pathways and various reconstructive procedures are site specific, and drainage occurs in a predictable manner^12^. The NCCN guidelines document several risk factors for predicting poor survival: extranodal extension, neck lymph node metastasis, depth of tumor invasion, and histological grade^13–15^. Although survival did vary with different anatomic subsites of OSCC, the NCCN treatment guidelines did not account for these differences in predicting prognosis.

OSCC that arises in different anatomic subsites may become more advanced because the tissue adjacent to the tumor can be a conduit for tumor invasion directly into the muscle, bone, and neurovascular tissue or for regional or distant node metastasis^4,16^. Nair et al. reported differences in survival and in clinical and pathological features between tongue and buccal OSCC^17^. Other investigators have suggested that OSCC in different subsites could, on the basis of various outcomes, be regarded as clinicopathologically distinct entities^18–20^. In those studies, the common problem was that OSCCs in some anatomic subsites are rare and thus difficult to investigate.

The aim of this study was to investigate whether different anatomical subsites of OSCC predict various survival conditions for patients with OSCC and to provide clinicians with information on the non-negligible differences in clinical and pathological characteristics among the different subsites. Since lip OSCC is also highly associated with betel nut chewing in Taiwan, we included patients with lip OSCC^21^.

## Results

We analyzed data from 3010 patients. The dominant OSCC site was the buccal mucosa (n = 1050, 34.9%), followed by the tongue (n = 884, 29.4%), and alveolar ridge (n = 482, 16%). Table 1 presents the clinicopathological characteristics of OSCCs at different subsites. Of the patients, 2864 were male and 146 were female. The age at diagnosis of the tumors mainly ranged from 51 to 60 years (35.4%) and from 41 to 50 years (22.5%), respectively and the same trend could be seen in the OSCCs occurred in the buccal mucosa, tongue, floor of the mouth, and retromolar trigone (*p* < 0.001). Additionally, the age at diagnosis of the other subsites of OSCCs mainly ranged from 51 to 60 years and followed by 61 to 70 years, respectively. Pathological T stage I, II, III, and IV diseases were found in 1320 (43.9%), 722 (24%), 173 (5.7%), and 795 patients (26.4%), respectively. Pathological N negative disease was found in 1700 patients (56.5%), and pathological N stage I, II, and III diseases were found in 223 (7.4%), 394 (13.1%), and 29 patients (1.0%), respectively. In terms of pathological features, extracapsular nodal spread (ECS) and neck lymph node skip metastasis were observed in 233 (7.7%) and 53 patients (1.8%), respectively. In terms of pathological grade, 514 patients (17.5%) had well-differentiated OSCCs, whereas 2269 (77.5%) and 146 patients (5.0%) had moderately differentiated and poorly differentiated SCCs, respectively.

**Table 1:** The clinicopathological characteristics of cases by anatomic location.

	Total (n = 3010)		Anatomic site																p value
			Buccal mucosa (n = 1050)		Alveolar ridge (n = 482)		Ant tongue (n = 884)		Hard palate (n = 87)		Floor of mouth (n = 85)		RMT (n = 158)		Mucosal lip (n = 73)		Body of the lip (n = 191)		
	N	%	N	%	N	%	N	%	N	%	N	%	N	%	N	%	N	%	
**Gender**																			
Female	146	4.9	24	2.3	21	4.4	80	9.0	1	1.1	4	4.7	3	1.9	2	2.7	11	5.8	< 0.001
Male	2864	95.1	1026	97.7	461	95.6	804	91.0	86	98.9	81	95.3	155	98.1	71	97.3	180	94.2	
**Age**																			
≤ 40	201	6.7	73	7.0	13	2.7	95	10.7	0	0.0	3	3.5	12	7.6	0	0.0	5	2.6	< 0.001
41–50	677	22.5	232	22.1	89	18.5	229	25.9	10	11.5	26	30.6	43	27.2	13	17.8	35	18.3	
51–60	1067	35.4	394	37.5	171	35.5	285	32.2	41	47.1	34	40.0	58	36.7	24	32.9	60	31.4	
61–70	684	22.7	227	21.6	131	27.2	181	20.5	25	28.7	18	21.2	26	16.5	25	34.2	51	26.7	
≥ 71	381	12.7	124	11.8	78	16.2	94	10.6	11	12.6	4	4.7	19	12.0	11	15.1	40	20.9	
**Smoking**																			
No	589	19.6	199	19.0	86	17.8	184	20.8	18	20.7	12	14.1	25	15.8	14	19.2	51	26.7	0.126
Yes	2421	80.4	851	81.0	396	82.2	700	79.2	69	79.3	73	85.9	133	84.2	59	80.8	140	73.3	
**Betel nut**																			
No	970	32.2	335	31.9	142	29.5	307	34.7	29	33.3	23	27.1	41	25.9	22	30.1	71	37.2	0.162
Yes	2040	67.8	715	68.1	340	70.5	577	65.3	58	66.7	62	72.9	117	74.1	51	69.9	120	62.8	
**Alcohol**																			
No	1014	33.7	367	35.0	175	36.3	285	32.2	28	32.2	18	21.2	40	25.3	26	35.6	75	39.3	0.018
Yes	1996	66.3	683	65.0	307	63.7	599	67.8	59	67.8	67	78.8	118	74.7	47	64.4	116	60.7	
**T stage**																			
1	1320	43.9	487	46.4	136	28.2	398	45.0	39	44.8	47	55.3	47	29.7	45	61.6	121	63.4	< 0.001
2	722	24.0	282	26.9	66	13.7	233	26.4	16	18.4	21	24.7	35	22.2	19	26.0	50	26.2	
3	173	5.7	78	7.4	8	1.7	60	6.8	6	6.9	1	1.2	7	4.4	4	5.5	9	4.7	
4	795	26.4	203	19.3	272	56.4	193	21.8	26	29.9	16	18.8	69	43.7	5	6.8	11	5.8	
**N stage**																			
Without ND	664	22.1	228	21.7	118	24.5	154	17.4	31	35.6	13	15.3	30	19.0	19	26.0	71	37.2	< 0.001
0	1700	56.5	605	57.6	271	56.2	491	55.5	42	48.3	55	64.7	91	57.6	45	61.6	100	52.4	
1	223	7.4	86	8.2	24	5.0	77	8.7	7	8.0	5	5.9	9	5.7	3	4.1	12	6.3	
2	394	13.1	121	11.5	64	13.3	150	17.0	7	8.0	11	12.9	27	17.1	6	8.2	8	4.2	
3	29	1.0	10	1.0	5	1.0	12	1.4	0	0.0	1	1.2	1	0.6	0	0.0	0	0.0	
**Overall stage**																			
Early	1766	58.7	660	62.9	187	38.8	530	60.0	49	56.3	59	69.4	66	41.8	59	80.8	156	81.7	< 0.001
Advance	1244	41.3	390	37.1	295	61.2	354	40.0	38	43.7	26	30.6	92	58.2	14	19.2	35	18.3	
**ECS**																			
Without ND	664	22.1	228	21.7	118	24.5	154	17.4	31	35.6	13	15.3	30	19.0	19	26.0	71	37.2	< 0.001
No	2113	70.2	739	70.4	334	69.3	648	73.3	51	58.6	63	74.1	115	72.8	50	68.5	113	59.2	
Yes	233	7.7	83	7.9	30	6.2	82	9.3	5	5.7	9	10.6	13	8.2	4	5.5	7	3.7	
**Neck LV IV and V metastasis**																			
No	2937	97.6	1030	98.1	473	98.1	854	96.6	84	96.6	83	97.6	151	95.6	72	98.6	190	99.5	0.112
Yes	73	2.4	20	1.9	9	1.9	30	3.4	3	3.4	2	2.4	7	4.4	1	1.4	1	0.5	
**Skip metastasis**																			
No	2957	98.2	1040	99.0	478	99.2	852	96.4	86	98.9	83	97.6	154	97.5	73	100.0	191	100.0	< 0.001
Yes	53	1.8	10	1.0	4	0.8	32	3.6	1	1.1	2	2.4	4	2.5	0	0.0	0	0.0	
**Grade**																			
Well	514	17.5	220	21.5	79	16.8	113	13.3	20	23.3	4	4.8	18	11.9	15	20.5	45	23.9	< 0.001
Moderately	2269	77.5	765	74.6	371	78.9	683	80.2	62	72.1	74	88.1	125	82.8	56	76.7	133	70.7	
Poorly	146	5.0	40	3.9	20	4.3	56	6.6	4	4.7	6	7.1	8	5.3	2	2.7	10	5.3	
**Reccur**																			
No	2353	78.2	829	79.0	374	77.6	676	76.5	69	79.3	69	81.2	112	70.9	63	86.3	161	84.3	0.044
Yes	657	21.8	221	21.0	108	22.4	208	23.5	18	20.7	16	18.8	46	29.1	10	13.7	30	15.7	
**Death**																			
No	1781	59.2	682	65.0	249	51.7	513	58.0	34	39.1	52	61.2	82	51.9	52	71.2	117	61.3	< 0.001
Yes	1229	40.8	368	35.0	233	48.3	371	42.0	53	60.9	33	38.8	76	48.1	21	28.8	74	38.7	

*ND* neck dissection, *RMT* retromolar trigone, *ECS* extra-nodal spread.

Comparison of the patients according to anatomical subsites revealed that those whose primary tumor was at the FOM appeared to have greater association with alcohol consumption. Pathological T4 stage OSCCs occurred in the alveolar ridge and retromolar trigone in 56.4% and 43.7% of the patients, respectively. Further, > 25% of the patients with tongue OSCC and 23.4% of those with retromolar OSCC had pathological lymph node metastases. Moreover, lymph node skip metastases were highly likely to occur with tongue OSCC. More details of these differences are presented in Table 1.

The relationships between clinicopathological characteristics and recurrence or death are presented in Table 2. Age at diagnosis was significantly associated with cancer-related death (*p* < 0.001), and the patients aged > 71 years had the highest mortality (53.8%). Regarding pathological stages, those with advanced T and N stages presented with worse prognosis (both *p* < 0.001 in terms of recurrence and death). In terms of pathological features, ECS (both *p* < 0.001), level IV or V positive lymph nodes (*p* = 0.009 and *p* < 0.001, respectively), and pathological grade (both *p* < 0.001) were significantly associated with worse prognosis.

**Table 2:** Results of univariable analysis for cancer recurrence or death among patients.

	Total (n = 3010)		Recur		p value	Death		p value
			Yes (n = 657)			Yes (n = 1229)		
	N	%	N	%		N	%	
**Gender**								
Female	146	4.9	34	23.3	0.661	60	41.1	0.947
Male	2864	95.1	623	21.8		1169	40.8	
**Age**								
≤ 40	201	6.7	48	23.9	0.044	68	33.8	< 0.001
41–50	677	22.5	175	25.8		279	41.2	
51–60	1067	35.4	218	20.4		413	38.7	
61–70	684	22.7	136	19.9		264	38.6	
≥ 71	381	12.7	80	21.0		205	53.8	
**Smoking**								
No	589	19.6	145	24.6	0.068	246	41.8	0.607
Yes	2421	80.4	512	21.1		983	40.6	
**Betel nut**								
No	970	32.2	229	23.6	0.103	403	41.5	0.582
Yes	2040	67.8	428	21.0		826	40.5	
**Alcohol**								
No	1014	33.7	239	23.6	0.099	407	40.1	0.582
Yes	1996	66.3	418	20.9		822	41.2	
**T stage**								
1	1320	43.9	243	18.4	< 0.001	404	30.6	< 0.001
2	722	24.0	168	23.3		299	41.4	
3	173	5.7	40	23.1		83	48.0	
4	795	26.4	206	25.9		443	55.7	
**N stage**								
Without ND	664	22.1	164	24.7	< 0.001	277	41.7	< 0.001
0	1700	56.5	276	16.2		566	33.3	
1	223	7.4	63	28.3		114	51.1	
2	394	13.1	142	36.0		258	65.5	
3	29	1.0	12	41.4		14	48.3	
**Stage**								
Early	1766	58.7	328	18.6	< 0.001	573	32.4	< 0.001
Advance	1244	41.3	329	26.4		656	52.7	
**ECS**								
Without ND	664	22.1	164	24.7	< 0.001	277	41.7	< 0.001
No	2113	70.2	404	19.1		801	37.9	
Yes	233	7.7	89	38.2		151	64.8	
**Neck LV IV and V metastasis**								
No	2937	97.6	632	21.5	0.009	1182	40.2	< 0.001
Yes	73	2.4	25	34.2		47	64.4	
**Contralateral neck metastasis**								
No	3004	99.8	655	21.8	0.495	1227	40.8	0.708
**Skip metastasis**								
Yes	6	0.2	2	33.3		2	33.3	
No	2957	98.2	639	21.6	0.031	1203	40.7	0.219
Yes	53	1.8	18	34.0		26	49.1	
**Close margin**								
No	2898	96.3	636	21.9	0.422	1180	40.7	0.522
Yes	112	3.7	21	18.8		49	43.8	
**Grade**								
Well	514	17.5	76	14.8	< 0.001	136	26.5	< 0.001
Moderately	2269	77.5	512	22.6		968	42.7	
Poorly	146	5.0	47	32.2		86	58.9	
**Anatomic site**								
Buccal mucosa	1050	34.9	221	21.0	0.044	368	35.0	< 0.001
Alveolar ridge	482	16.0	108	22.4		233	48.3	
Ant tongue	884	29.4	208	23.5		371	42.0	
Hard palate	87	2.9	18	20.7		53	60.9	
Floor of mouth	85	2.8	16	18.8		33	38.8	
RMT	158	5.2	46	29.1		76	48.1	
Mucosal lip	73	2.4	10	13.7		21	28.8	
Body of the lip	191	6.3	30	15.7		74	38.7	
**Recur**								
No	2353	78.2				734	31.2	< 0.001
Yes	657	21.8				495	75.3	
**Dead**								
No	1781	59.2	162	9.1	< 0.001			
Yes	1229	40.8	495	40.3				

When the patients were compared according to anatomical subsite, OSCC arising from the retromolar trigone appeared to be more related to recurrence, and OSCC arising from the hard palate appeared to be more closely associated with cancer-related death (*p* = 0.044 and *p* < 0.001, respectively).

The results of the univariate and multivariate Cox regression analyses for disease-free survival (Table 3) revealed that the patients with OSCCs in the hard palate and alveolar ridge had the poorest disease-free survival outcomes, with the hazard ratios of 1.848 (*p* < 0.001) and 1.22 (*p* = 0.017), respectively.

**Table 3:** Multiple Cox proportional hazards regression analysis of disease-free survival.

Cox proportional-hazards regression analysis of disease free survival									
	Total	Recur or death		Univariate analysis (crude)			Multiple analysis (adjusted)		
		N	%	Hazard ratio	95% CI	p value	Hazard ratio	95% CI	p value
**Age**									
≤ 40	201	82	40.8	1.000			1.000		
41–50	677	316	46.7	1.228	0.963–1.566	0.097	1.315	1.029–1.680	0.029
51–60	1067	470	44.0	1.167	0.923–1.476	0.196	1.256	0.990–1.594	0.060
61–70	684	306	44.7	1.239	0.971–1.582	0.085	1.426	1.113–1.829	0.005
≥ 71	381	217	57.0	1.712	1.327–2.208	< 0.001	2.042	1.574–2.648	< 0.001
**Anatomic subsite**									
Buccal mucosa	1050	441	42.0	1.000			1.000		
Alveolar ridge	482	252	52.3	1.384	1.186–1.616	< 0.001	1.220	1.036–1.436	0.017
Ant tongue	884	413	46.7	1.164	1.018–1.331	0.027	1.133	0.990–1.297	0.069
Hard palate	87	55	63.2	1.877	1.418–2.485	< 0.001	1.848	1.394–2.450	< 0.001
Floor of mouth	85	38	44.7	1.174	0.843–1.636	0.343	1.185	0.850–1.652	0.317
RMT	158	85	53.8	1.331	1.055–1.679	0.016	1.190	0.941–1.504	0.147
Mucosal lip	73	24	32.9	0.834	0.553–1.258	0.386	0.918	0.608–1.386	0.683
Body of the lip	191	83	43.5	0.942	0.745–1.191	0.616	1.031	0.814–1.307	0.799
**T stage**									
1	1320	497	37.7	1.000			1.000		
2	722	342	47.4	1.395	1.215–1.601	< 0.001	1.271	1.105–1.462	0.001
3	173	88	50.9	1.715	1.367–2.152	< 0.001	1.487	1.181–1.871	0.001
4	795	464	58.4	1.904	1.678–2.161	< 0.001	1.585	1.379–1.823	< 0.001
**N positive**									
No	2364	992	42.0	1.000			1.000		
Yes	646	399	61.8	1.959	1.744–2.201	< 0.001	1.828	1.615–2.070	< 0.001

Follow-up time: from OSCC diagnosed to recur, death, or 2019–12-31.

*RMT* retromolar trigone.

**Figure 1:**
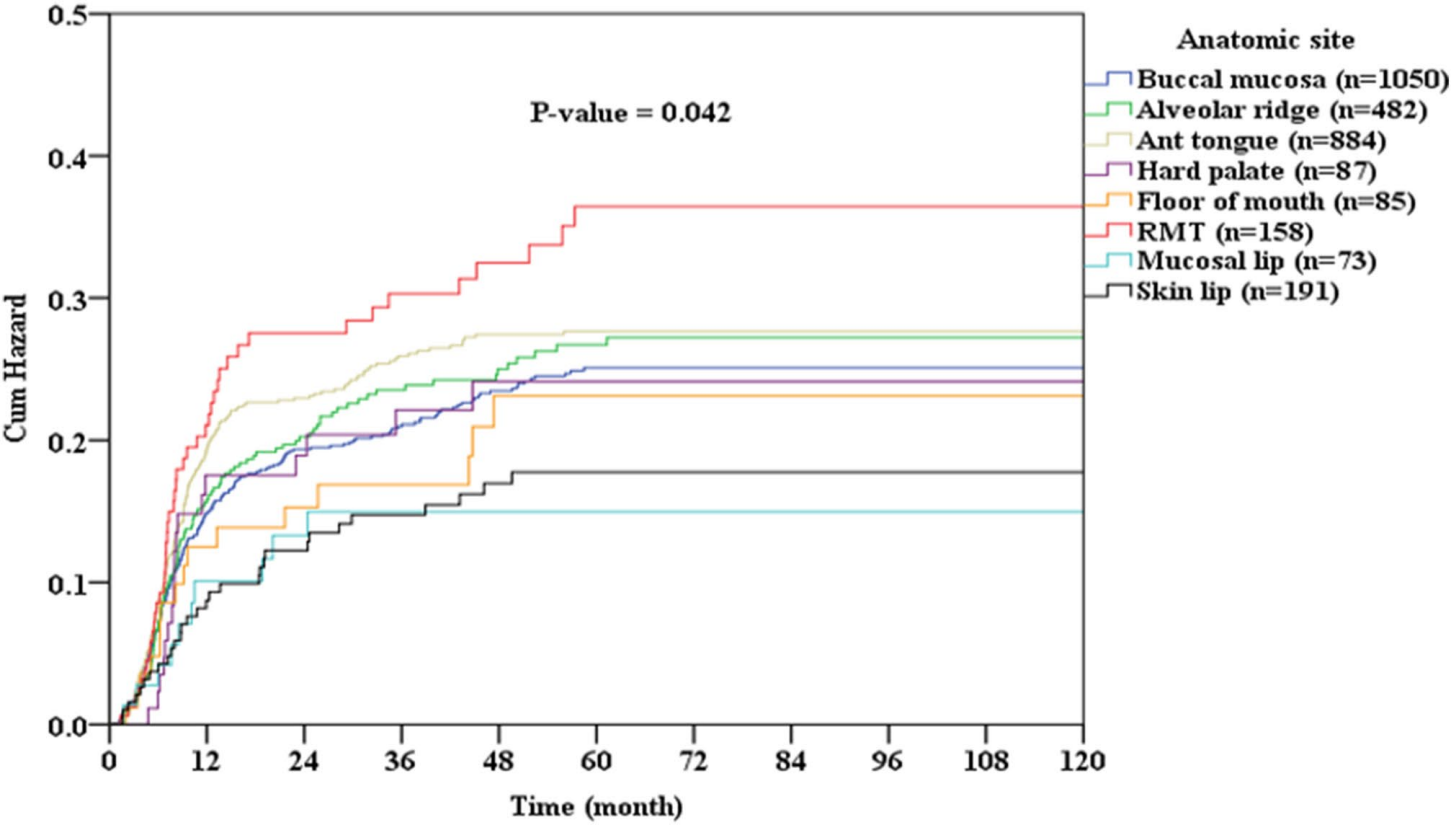
Cumulative recurrence rate oral cavity squamous cell carcinoma (OSCC) according to location of disease.

**Figure 2:**
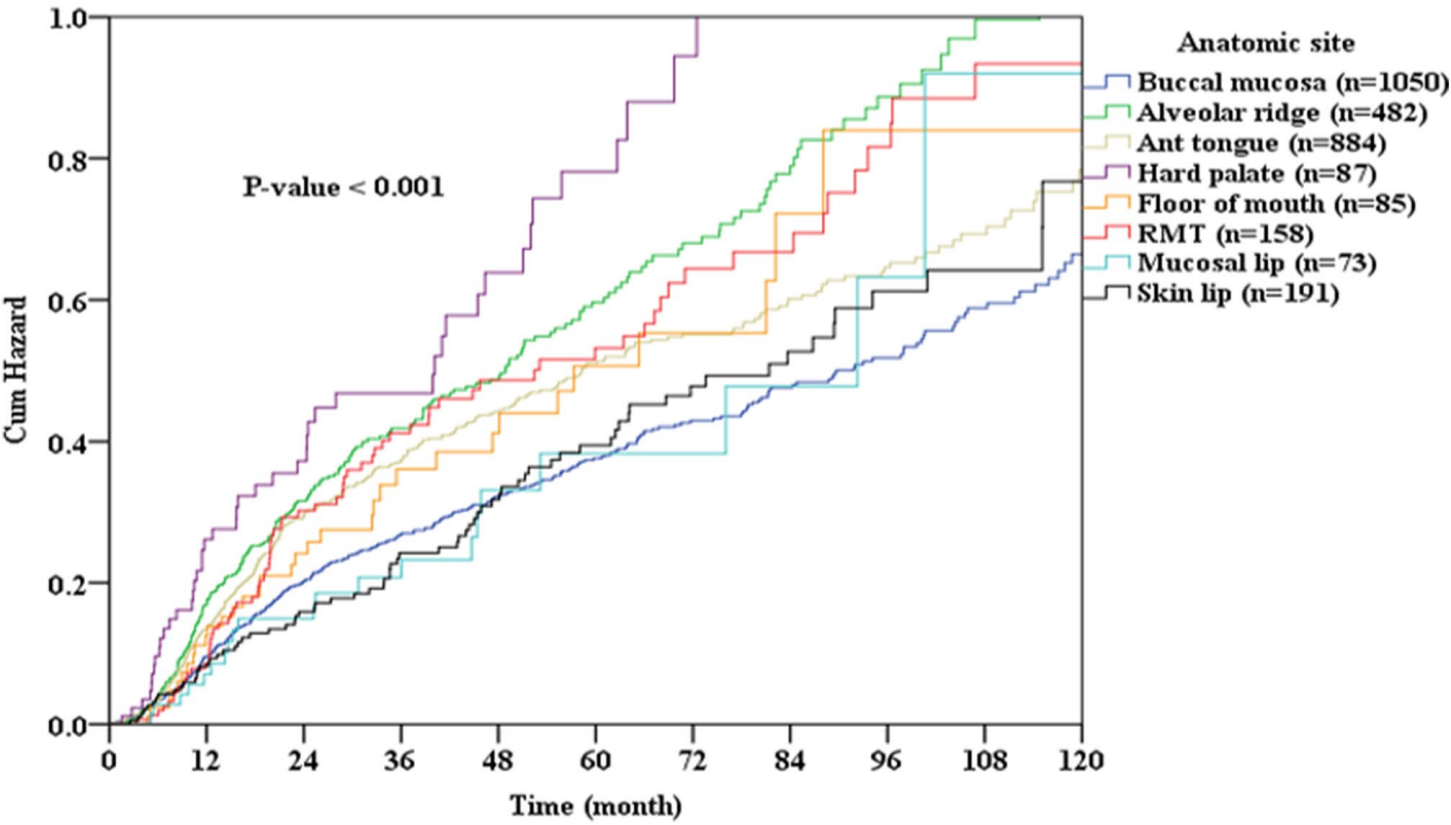
Cumulative rate of cancer-related death among patients with oral cavity squamous cell carcinoma (OSCC) according to location of disease.

Figure 1 shows the cumulative rates of OSCC recurrence according to the different subsites. OSCC that arose from the retromolar trigone, tongue, and alveolar ridge had the highest rates of recurrence, and those of the lip mucosa and body of the lip had the lowest rates of recurrence (*p* = 0.042). Figure 2 shows the cumulative rates of cancer-related death among patients according to different subsites of OSCC. OSCC that arose from the hard palate, alveolar ridge, and retromolar trigone caused the highest rates of death, and those of the body of the lip, buccal mucosa, and lip mucosa caused the lowest rates of death (*p* < 0.001).

## Discussion

From the perspective of mortality, Farhood et al. reviewed data for 20,647 patients from the Surveillance, Epidemiology, and End Results Program (SEER) 9 database and reported that OSCC was most commonly diagnosed in the FOM and the tongue^19^. Furthermore, they found that tongue OSCC was associated with more cause-specific mortality than were OSCCs at other subsites^19^. Different cultures and dietary habits could account for the difference in the predilection site between Taiwan and other places^22,23^, and this could be the major reason for the difference between their results and ours. However, rates of survival among patients with OSCC in different subsites did differ, and NCCN treatment guidelines do not take this point into account. Su et al. studied a large Taiwanese population and reported that the 5-year rate of survival was poorest among patients with hard palate OSCC, followed by those with gingival and FOM OSCCs, and gingival and hard palate OSCCs were most likely to be diagnosed at an advanced stage^18^. They pointed out that OSCC at different subsites necessitated specific surveillance strategies and tailored treatment. Our results were consistent with theirs; however, we subclassified gingival OSCC as retromolar trigone and buccal mucosa OSCC according to the NCCN treatment guideline and found that survival and recurrence rates of the two did indeed differ. Second, our data obtained were from a single center, and all our patients underwent surgery and adjuvant therapy, performed by a single team of head and cancer specialists. Finally, apart from overall survival, we also found different recurrence rates among OSCCs at different subsites.

In our study, the risk of lymph node skip metastases was greatest with tongue OSCCs (3.6%), followed by retromolar trigone and FOM OSCCs. According to several reports, supra-omohyoid neck dissection was not sufficient for OSCC, and neck dissection at level IV should be routine to prevent neck metastasis^24,25^. However, other investigators have reported conflicting opinions^26^. Warshavsky et al. conducted a meta-analysis about the rationality of prophylactic level IV neck dissection for OSCC and reported that the rate of skip metastasis ranged from 0 to 5.50% with a fixed-effects model of 0.50% (95% confidence interval 0.09–1.11%), and staging and subsites of OSCC did not notably affect the rate of skip metastasis^27^. Weiss Shabtay and Ronen also conducted a meta-analysis about prophylactic level IV neck dissection for tongue OSCC and reported a 2.8% rate of skip metastasis^28^. In our study, the rate of lymph node skip metastasis in tongue cancer was 3.6%, which was consistent with Warshavsky et al.’s meta-analysis. Warshavsky et al. did not recommend prophylactic level IV neck dissection for tongue OSCC, but in clinical practice, surgeons must be aware that skip metastasis can occur with tongue OSCC.

From the perspective of recurrence, Liu et al. analyzed data from 1383 patients and reported that 489 patients (35.4%) developed recurrence^20^. Furthermore, OSCC most likely recurred in the retromolar trigone, followed by the FOM^20^. Nair et al. conducted a retrospective study that included 735 patients with tongue OSCC and 665 patients with buccal mucosa OSCC and reported that the former had more recurrence factors, including perineural invasion, lymphovascular emboli, and poorer pathological grade^17^. In our study, retromolar trigone OSCC was most likely to recur, followed by tongue, alveolar ridge, FOM. Compared with the previous studies, our study was conducted with a larger sample size and included all American Joint Committee on Cancer (AJCC) anatomic subsites in the analysis.

Hard palate and alveolar ridge OSCCs account for a small percentage of OSCCs in comparison with OSCCs in other subsites^29–31^. We found that hard palate and alveolar ridge OSCCs carried a higher risk of mortality than OSCCs at the other subsites. This finding has several explanations: OSCCs adjacent to the mandibular or maxillary bone could be more likely to be at an advanced T stage when diagnosed, and > 40% of both hard palate and alveolar ridge OSCCs occurred in patients aged > 60 years; age was an independent factor of disease-free survival in our study. Several investigators have reported that different strategies should be tailored to OSCCs at various subsites^17–19,32^, consistent with our findings.

From a biological perspective, different subsites of OSCC may have their own biomarkers, demonstrating discrepancies in survival and prognosis. Fu et al. reported that if patients with tongue and lip OSCC have high DEXD/H box helicase 60 (DDX60) expression, the outcome will be poor^33^. They concluded that DDX60 is a novel but subsite-specific biomarker for OSCC. Boldrup et al. also reported that patients with tongue OSCC demonstrate significant deregulation of miR-21, miR-125b, and miR-203, but those with gingival OSCC demonstrate significant downregulation of only miR-125b^34^. In the future, biomarkers for different subsites of OSCC should be analyzed to further explain the differences in survival among patients with OSCCs at all subsites.

Our study had several limitations. First, the retrospective study design may have contributed to bias. Second, the data for our study were collected from a single medical center in Taiwan. Oral cavity cancer is strongly associated with betel nut chewing, which is popular in Taiwan; therefore, our results may differ from findings in other geographical regions^5,35^. Finally, in our result, the tissue adjacent to the metastatic patterns of the tumor and lymph nodes cannot fully explain why the patients with hard palate OSCC had the worst disease-free survival and why alveolar ridge OSCC was most likely to recur. Further investigations may focus on discrepancies among biomarkers for OSCCs at different subsites.

In conclusion, survival distinctly differed among patients with OSCCs at different subsites, although the NCCN treatment guideline did not account for subsites of OSCC. Our results not only were consistent with the findings of previous studies but also may encourage future investigations on Taiwanese patients with OSCC at different subsites.

## Material and methods

### Patients

This retrospective cohort study was approved by the institutional review board and ethics committee of Changhua Christian Hospital, Changhua, Taiwan (IRB No. 210210). We obtained all clinical data through a chart review and the cancer registry center of Changhua Christian Hospital. We confirmed that all the methods were performed in accordance with relevant guidelines and regulations. Informed consent was waived owing to the retrospective nature of the study, and the analysis used anonymized clinical data with the approval of the IRB of Changhua Christian Hospital, Changhua, Taiwan (IRB No. 210210). We identified 3620 patients with OSCC who underwent surgery, adjuvant therapy, and follow-up at our center between January 1, 2008, and December 31, 2018. The follow-up duration was from the initial date of diagnosis to December 31, 2019. We excluded patients who did not receive treatment in accordance with the NCCN cancer treatment guidelines, whose initial diagnosis at our hospital was recurrence or distant metastasis, and who did not undergo surgery at our hospital. In total, 3010 patients were enrolled in our study and assigned into subgroups according to the following pathological anatomical sites designated by the AJCC: the buccal mucosa, alveolar ridge, anterior tongue, hard palate, FOM, retromolar trigone, mucosa of the lip, and body of the lip.

### Treatment protocols

The patients enrolled in our study underwent wide tumor excision and neck dissection according to clinical tumor stage. The patients with clinical stage N0 tumors underwent selective neck dissection, and those with N-positive tumors underwent radical neck dissection. Adjuvant therapy was performed in individual cases by our interdisciplinary head and neck surgery team, which included surgeons, oncology radiologists, a medical oncologist, and a pathologist. In general, postoperative radiotherapy was administered to patients with pathological T3 or T4 primary tumors, N2 and N3 stage nodal disease, N1 stage at levels IV or V, vascular embolism, or perineural invasion, as determined in the final pathological specimens. In our hospital, radiotherapy is considered for patients with one positive node or perineural invasion who do not exhibit other adverse features. Postoperative radiochemotherapy was administered in patients with ECS and positive margins. Radiochemotherapy can also be considered for patients with pT3 or pT4 primary tumors, N2 or N3 stage nodal disease, nodal disease at levels IV or V, perineural invasion, or vascular embolism. Radiotherapy was administered no more than 6 weeks after surgery and was delivered by a linear accelerator at a total dose of 60–66 Gy (1.8–2.0 Gy/fraction). If chemotherapy concurrent with radiotherapy was indicated, cisplatin (80 mg/m^2^) and 5-fluorouracil (400–500 mg/m^2^) were administered in two cycles and repeated after 4–5 weeks. The treatment protocol used in this study was previously described^36^.

### Clinical and pathological parameters

We recorded the patients’ sex, age at OSCC diagnosis, survival time, pathological AJCC anatomical site, AJCC (7th edition) TNM stage, pathological grade, recurrence, and positive lymph nodes at each level of the neck. Skip metastasis was defined as positive neck metastasis at levels IV and V without the involvement of higher levels (levels I–III). We also recorded behaviors such as smoking, chewing betel nuts, and alcohol consumption. The anatomical sites were then subclassified as the alveolar ridge, anterior two-thirds of the tongue, buccal mucosa, hard palate, FOM, retromolar trigone, mucosa of the lip, and body of the lip. Information about mortality was retrieved from the cancer registry center of Changhua Christian Hospital and from data updated annually by the Health Bureau of Changhua City.

### Statistical analyses

We calculated continuous and categorical variables as mean ± standard deviation and percentage, respectively. We used the Mann–Whitney *U* test to compare the continuous variables and the chi-square test to compare the differences in the categorical variables among the different patient groups. To examine the effects of the clinicopathological factors on survival in patients with OSCCs, we used univariate and multivariate Cox proportional hazards models. We subsequently calculated hazard ratios and 95% confidence intervals. Rates of outcomes were estimated using Kaplan–Meier analyses. To compare the group survival functions, we used log-rank tests based on survival data. A *p* value of < 0.05 was considered statistically significant. To perform all statistical analyses, we used the statistical package SPSS version 16 for Windows (SPSS, Chicago, IL, USA).

## Data Availability

The datasets generated during and/or analysed during the current study are available from the corresponding author on reasonable request.

## References

[1] Ghantous Y, Abu Elnaaj I (2017). Global incidence and risk factors of oral cancer. Harefuah.

[2] Peres MA (2019). Oral diseases: A global public health challenge. Lancet.

[3] Thompson L (2006). World Health Organization classification of tumours: Pathology and genetics of head and neck tumours. Ear Nose Throat J..

[4] Shah JP, Gil Z (2009). Current concepts in management of oral cancer-surgery. Oral Oncol..

[5] Kao SY, Lim E (2015). An overview of detection and screening of oral cancer in Taiwan, China. Chin. J. Dent. Res..

[6] Yang YH, Warnakulasuriya S, Yang HF, Lin LJ, Wang YW (2020). Public health measures to reduce areca nut and betel quid use for control of oral cancer in Taiwan. Oral Oncol..

[7] Su SY, Chen WT, Chiang CJ, Yang YW, Lee WC (2020). Oral cancer incidence rates from 1997 to 2016 among men in Taiwan: Association between birth cohort trends and betel nut consumption. Oral Oncol..

[8] Lin YS, Jen YM, Wang BB, Lee JC, Kang BH (2005). Epidemiology of oral cavity cancer in Taiwan with emphasis on the role of betel nut chewing. ORL J. Otorhinolaryngol. Relat. Spec..

[9] Chang KM (1966). Betel nut chewing and mouth cancer in Taiwan. 2. Observation of the oral mucosa in the betel nut chewer. Taiwan Yi Xue Hui Za Zhi.

[10] Colevas AD (2018). NCCN guidelines insights: Head and neck cancers, version 1.2018. J. Natl. Compr. Canc. Netw..

[11] Adelstein D (2017). NCCN guidelines insights: Head and neck cancers, version 2.2017. J. Natl. Compr. Canc. Netw..

[12] Mukherji SK, Armao D, Joshi VM (2001). Cervical nodal metastases in squamous cell carcinoma of the head and neck: What to expect. Head Neck..

[13] Caldeira PC, Soto AML, de Aguiar MCF, Martins CC (2020). Tumor depth of invasion and prognosis of early-stage oral squamous cell carcinoma: A meta-analysis. Oral Dis..

[14] Huang SH, O’Sullivan B (2017). Overview of the 8th edition TNM classification for head and neck cancer. Curr. Treat. Options Oncol..

[15] Ettinger KS, Ganry L, Fernandes RP (2019). Oral cavity cancer. Oral Maxillofac. Surg. Clin. N. Am..

[16] Trotta BM, Pease CS, Rasamny JJ, Raghavan P, Mukherjee S (2011). Oral cavity and oropharyngeal squamous cell cancer: Key imaging findings for staging and treatment planning. Radiographics.

[17] Nair S (2016). Squamous cell carcinoma of tongue and buccal mucosa: Clinico-pathologically different entities. Eur. Arch. Otorhinolaryngol..

[18] Su WW (2019). Impact of varying anatomic sites on advanced stage and survival of oral cancer: 9-year prospective cohort of 27 717 cases. Head Neck..

[19] Farhood Z, Simpson M, Ward GM, Walker RJ, Osazuwa-Peters N (2019). Does anatomic subsite influence oral cavity cancer mortality? A SEER database analysis. Laryngoscope.

[20] Liu SA (2017). Pathological features and their prognostic impacts on oral cavity cancer patients among different subsites—A singe institute’s experience in Taiwan. Sci. Rep..

[21] Tseng HW, Liou HH, Tsai KW, Ger LP, Shiue YL (2017). Clinicopathological study of lip cancer: A retrospective hospital-based study in Taiwan. APMIS.

[22] Liao CT (2010). Tongue and buccal mucosa carcinoma: Is there a difference in outcome?. Ann. Surg. Oncol..

[23] Krishna Rao SV, Mejia G, Roberts-Thomson K, Logan R (2013). Epidemiology of oral cancer in Asia in the past decade–an update (2000–2012). Asian Pac. J. Cancer Prev..

[24] Byers RM (1997). Frequency and therapeutic implications of “skip metastases” in the neck from squamous carcinoma of the oral tongue. Head Neck..

[25] De Zinis LO, Bolzoni A, Piazza C, Nicolai P (2006). Prevalence and localization of nodal metastases in squamous cell carcinoma of the oral cavity: Role and extension of neck dissection. Eur. Arch. Otorhinolaryngol..

[26] Crean SJ, Hoffman A, Potts J, Fardy MJ (2003). Reduction of occult metastatic disease by extension of the supraomohyoid neck dissection to include level IV. Head Neck..

[27] Warshavsky A (2019). Assessment of the rate of skip metastasis to neck Level IV in patients with clinically node-negative neck oral cavity squamous cell carcinoma: A systematic review and meta-analysis. JAMA Otolaryngol. Head Neck Surg..

[28] Weisz Shabtay N, Ronen O (2020). Level IV neck dissection as an elective treatment for oral tongue carcinoma-a systematic review and meta-analysis. Oral Surg. Oral Med. Oral Pathol. Oral Radiol..

[29] Alonso JE (2018). The survival impact of surgical therapy in squamous cell carcinoma of the hard palate. Laryngoscope.

[30] Obayemi A (2019). Elective neck dissection (END) and cN0 hard palate and upper gingival cancers: A National Cancer Database analysis of factors predictive of END and impact on survival. J. Surg. Oncol..

[31] Eskander A (2013). Outcome predictors in squamous cell carcinoma of the maxillary alveolus and hard palate. Laryngoscope.

[32] de Boer MF, Sanderson RJ, Damhuis RA, Meeuwis CA, Knegt PP (1997). The effects of alcohol and smoking upon the age, anatomic sites and stage in the development of cancer of the oral cavity and oropharynx in females in the south west Netherlands. Eur. Arch. Otorhinolaryngol..

[33] Fu TY (2016). Subsite-specific association of DEAD box RNA helicase DDX60 with the development and prognosis of oral squamous cell carcinoma. Oncotarget.

[34] Boldrup L, Coates PJ, Wahlgren M, Laurell G, Nylander K (2012). Subsite-based alterations in miR-21, miR-125b, and miR-203 in squamous cell carcinoma of the oral cavity and correlation to important target proteins. J. Carcinog..

[35] Chuang SL (2017). Population-based screening program for reducing oral cancer mortality in 2,334,299 Taiwanese cigarette smokers and/or betel quid chewers. Cancer.

[36] Lin NC, Su IH, Hsu JT, Chang YJ, Tsai KY (2021). Comparison of different lymph node staging systems in patients with positive lymph nodes in oral squamous cell carcinoma. Oral Oncol..

